# Mixed methods scoping review of patients’ experiences of urgent and emergency cancer care

**DOI:** 10.1007/s00520-025-09245-8

**Published:** 2025-02-21

**Authors:** A. L. Hurley-Wallace, J. Defty, A. Richardson, R. Wagland

**Affiliations:** 1 University Hospital Southampton NHS Foundation Trust, Southampton, UK; 2 School of Health Sciences, Faculty of Life and Environmental Sciences, University of Southampton, Southampton, UK; 3 NIHR Applied Research Collaboration Wessex, Southampton, UK

**Keywords:** Acute oncology, Oncological emergencies, Patient experience, Scoping review urgent cancer care

## Abstract

**Background:**

Patients with oncological emergencies require immediate specialist cancer care to ensure optimum outcomes. This is often a stressful, time-sensitive situation for patients and their families who describe having to navigate complex care pathways to access urgent treatment. Acute oncology was established as a subspecialty in the UK in 2009, with the goal to streamline emergency cancer care. Patient experiences of urgent care in acute oncology contexts have not specifically been explored; hence, it is unknown whether patient experiences of emergency cancer care have improved. This may be due to lack of a patient-reported experience measure for this purpose.

**Methods:**

A mixed methods scoping review was conducted from 2010 to April 2024, with the aim of identifying core aspects of the care experience important to patients with cancer during an acute oncological episode, based on published research evidence. Qualitative, quantitative, and mixed methods articles were sourced and screened in accordance with Joanna Briggs Institute scoping review guidance. Core domains of patient experience were collated and summarised using mixed methods evidence synthesis.

**Results:**

Fifteen articles reporting on 14 unique studies were included. Issues important to patient experiences of emergency cancer care were described by patients, healthcare professional, and carer proxies. Studies captured experiences of various care delivery models including telehealth, in-person presentation to an Emergency Department, and acute oncology services. Six core domains of patient experience arose from the synthesis: managing emotional distress, being treated with compassion and respect, deciding when to seek help, hospital environment, quality of care and communication, and discharge planning.

**Conclusions:**

This is the first review to identify existing literature on patient experiences of emergency cancer care, highlighting core domains of patient experience impactful for patients and their families. Patients’ decisions about when to seek help and the impact of discussing palliative care needs during an emergency were more specific to emergency cancer care, compared to issues like the hospital environment, which can be important throughout a patient’s cancer care journey. Results will help inform development of a patient-reported experience measure to allow healthcare providers to evaluate and continuously improve specialist urgent and emergency cancer care services.

**Supplementary Information:**

The online version contains supplementary material available at 10.1007/s00520-025-09245-8.

## Background

Progress in cancer care has led to an increase in the number of patients receiving treatment in community settings. As a result, the number of patients presenting with cancer-related emergencies has increased [1, 2]. Acute oncological (AO) emergencies may be related to complications of cancer (e.g. malignant spinal cord compression, pulmonary embolism) or complications of treatment (e.g. neutropenic sepsis, life-threatening immunotherapy toxicities) [2]. Early recognition of these problems is essential.

Patients with cancer experiencing complications or treatment-related symptoms can be seriously ill, and in need of immediate specialist care and advice to prevent death, putting both patients and their families in stressful situations, often in an unfamiliar environment [3]. In 2008, a confidential enquiry in most of the United Kingdom (UK) and the British Crown Dependencies found this patient group received particularly poor quality care [4]. This led to the development of AO services in the UK and, over the past decade, different models of delivering specialist emergency cancer care have emerged internationally [5]. AO integrates knowledge and skills from emergency care, acute medicine, and palliative care, aiming to provide a streamlined service for people presenting with oncological emergencies [6].

In the UK, AO service provision is inconsistent, ranging from advisory services provided to admitting specialties (at district hospitals) to the delivery of 24-h specialist assessment units (at larger cancer centres) [7]. In contrast, AO across North America and Australasia has, historically, focused on preventative strategies, including patient education about treatment toxicities, telehealth assessments, and support, which are used frequently in countries with remote areas, such as Australia [5].

Despite the existence of AO services and strategies, many patients do not present or have direct access to these services during cancer-related emergencies. Instead, patients and their families often negotiate complex care pathways, for example via Emergency Departments (ED), to seek help [8, 9]. Variations in AO service provision substantially impact patients’ experiences of emergency cancer care, where patients report a disconnect between different components of the urgent and emergency care system, and poor cross-service communication regarding their cancer diagnosis and treatment [3]. This not only increases patient burden, but from a patient safety perspective, the increased time-to-treatment in an emergency can negatively impact patient outcomes [10].

Patient-reported experience measures (PREMs) are a well-established method used to measure healthcare service quality [11], which work by translating the patient voice into numerical data [10]. In the UK, patient-reported experience and outcomes are embedded within National Health Service (NHS) accountability frameworks and commissioning guidelines for AO services [12, 13]. Utilising PREMs ensures service improvements align with outcomes that matter most to patients and their families. Despite this, there is no measure available to capture patient experiences of emergency cancer care, resulting in a substantial knowledge gap, particularly which aspects of patients’ emergency cancer care experience have or have not improved since the inception of AO services.

An essential step towards developing a PREM is to understand which aspects of the care experience are most relevant to include as questionnaire items. Alongside patient consultation, generating items involves undertaking a literature review to collate patient experiences reported in research [14, 15]. This paper presents a mixed methods scoping review, with the aim of identifying core aspects of the care experience important to patients with cancer during an acute oncological episode. Objectives were the following:

1. Identify primary research studies that capture patient experiences of care during an acute oncological episode.
2. Summarise the experiences of patients who have sought medical advice or treatment for an acute oncological episode, via an urgent or emergency care service.
3. Inform the identification of a set of domains that encompass core issues important or impactful to patient experiences of care during an acute oncological episode.

## Methods

Scoping review procedures followed the Joanna Briggs Institute scoping review guide [16]. The review protocol was made publicly available on the Open Science Framework [17].

### Patient and public involvement

An advisory group including patient and public involvement representatives, AO clinicians, and representatives from the patient involvement team at University Hospital Southampton NHS Foundation Trust, Wessex Cancer Alliance, and the UK Acute Oncology Society, was consulted throughout. The advisory group was specifically formed to offer advice and feedback on this review, such as the review’s objectives, key search terms, and search strategy, as part of a larger research project (the PREMAC study) seeking to develop a PREM for AO services.

### Identification of articles

A search strategy and search terms for MEDLINE, CINAHL and PsycInfo (via EBSCO), Embase (via Ovid), and Web of Science (Clarivate™) databases were developed with assistance from an Academic Engagement Librarian at the University of Southampton. Databases were searched from 1 January 2010 to 22 April 2024, to capture literature published following the development of acute oncology as a speciality. Full search terms used are available in Additional file 1. Qualitative, quantitative, and mixed methods studies were included. Only articles written in English were included, due to resource constraints. Table 1 details full inclusion and exclusion criteria for articles in this review.

**Table 1:** Article inclusion and exclusion criteria

	Inclusion	Exclusion
Sample	• Patients with a cancer diagnosis, aged ≥ 18 years, who have experienced an acute oncological episode • Subsets of patients with cancer (e.g., emergency department sample with a subset of cancer patients) • Proxy reports of patient experience(s) from informal caregivers and/or healthcare professionals (HCPs)	• Participants < 18 years old/proxy report of patients < 18 years old • Non-cancer samples/samples with no cancer patient subset available • Non-emergent care samples
Context	• A primary study setting of acute care, including • urgent and emergency care services • Emergency and urgent care involving direct patient-healthcare professional interaction, delivered in any format (e.g., in-person, by telephone, etc.) • Studies aiming to capture the patient experience or record satisfaction with patient care • Published since 2010	• Studies that do not report the patient experience of care • Studies that only report patient experiences post-intervention or post-service improvement • Advice or care delivered without direct patient-healthcare professional interaction (i.e., automated advice from an e-health service)
Methodology	• Primary studies using any methodology (i.e., qualitative, quantitative, and mixed methods) • Study designs: cross sectional (including surveys), qualitative, pre-post, randomised controlled trials, non-randomised controlled trials (i.e., cluster and crossover trials), case–control and cohort studies • Full text available in English • Published in peer-reviewed academic journal	• Conference abstracts, posters, protocols, case reports, case series, letters to the editor, opinion papers, and commentaries • Secondary studies and reviews (systematic, narrative, scoping, and meta-analyses) Full-text unavailable in English

### Article screening

Duplicate articles were removed using EndNote™ 20 (Clarivate™). All articles were then imported into the systematic review platform Rayyan (QCRI) [18] for screening. Screening was conducted by AHW and RW, according to pre-established criteria.

Articles were blinded using Rayyan and screened independently by each reviewer, based on titles and abstracts (stage 1). The review was then unblinded, and the two reviewers met to discuss articles with conflicting decisions, reaching consensus on articles to be included for full-text review (stage 2). Once reviewers came to an agreement, full-text articles were imported and re-blinded. Once full-text review had been completed independently, articles were unblinded to reach consensus on articles to include as final.

### Mixed methods evidence synthesis

Qualitative and quantitative data that captured patient experience were initially extracted from each included article into Excel® (Microsoft®). Patient experience data were then collated and organised into domains (categories of interest). An evidence synthesis is presented to summarise these domains, as interpreted by the research team. Data were initially extracted and collated by AHW. Domains were triangulated and agreed with RW.

## Results

**Fig. 1:**
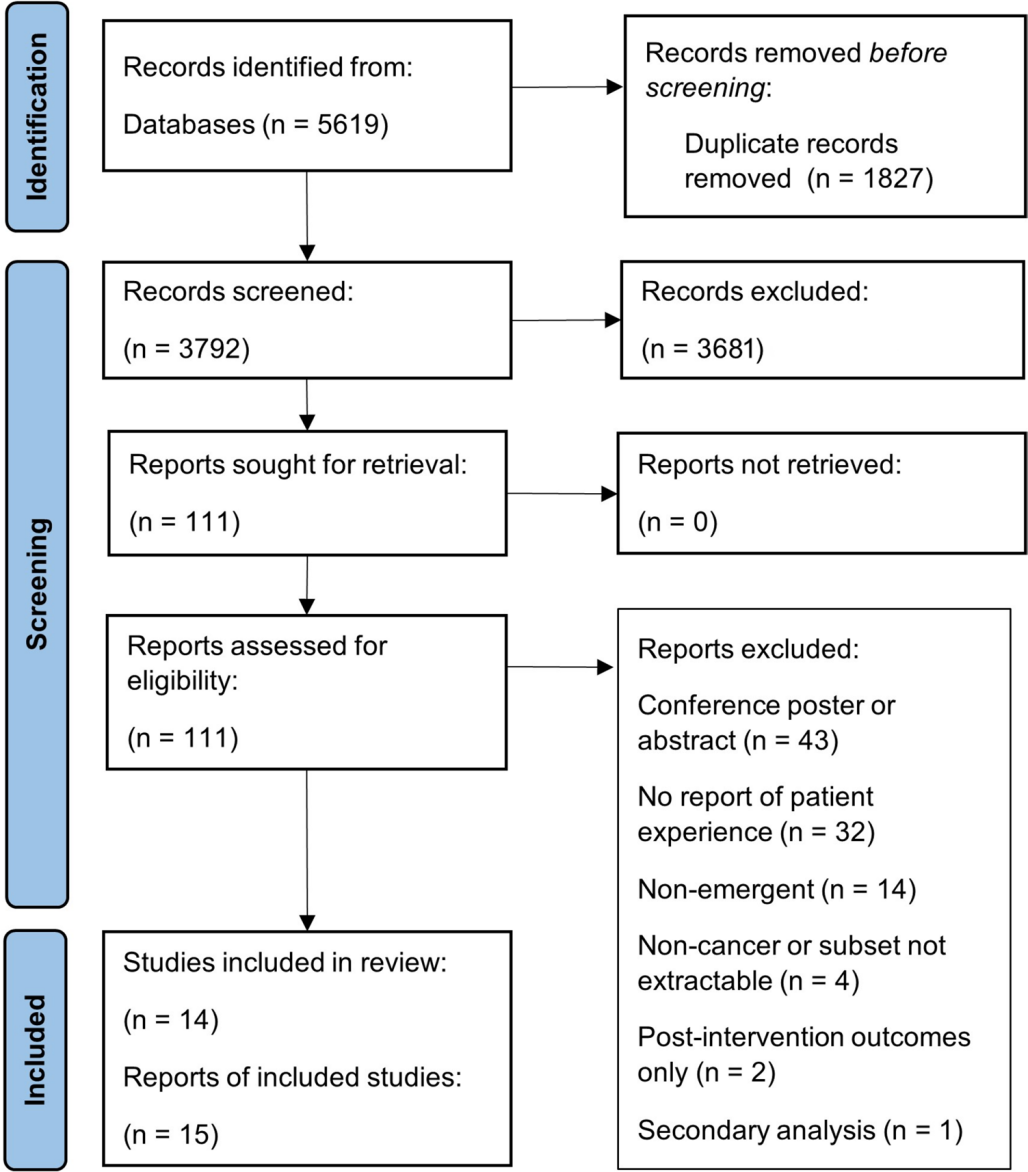
PRISMA flow diagram

Fifteen studies were included in the review. A PRISMA flow diagram (Fig. 1) shows the number of articles excluded at each stage.

Issues relevant to patient experiences of emergency cancer care were reported by patients themselves, as well as healthcare professional and carer proxies. Eight articles reported qualitative studies [19–26], four reported cross-sectional surveys [27–30], two were mixed methods [31, 32], and there was one before-and-after study that included patient opinion data [33].

Twelve articles reported experiences of cancer patients with mixed diagnoses (more than three cancer types in the sample) or did not specify diagnoses. The four remaining reports specified patients with brain cancer (*n* = 1) [30], lung cancer (*n* = 1) [19], solid tumours (*n* = 1) [24], and patients with metastatic, lung, or gastrointestinal cancer (*n* = 1) [33]. Details and key findings from included articles are summarised in Table 2.

**Table 2:** Summary of included articles (*n* = 15)

Authors (date), country	Study type	Participant type (*n*)	Patient age (years)	Cancer type	Setting (access route)	Primary aim/objectives	Summary of findings
Bailey et al. (2016), UK	Qualitative	Patients (*n* = 24), carers (*n* = 20) and clinicians (*n* = 50)	Age range 55–90	Lung cancer	Emergency department (ED)	To explore the experiences of patients with advanced Chronic Obstructive Pulmonary Disease and lung cancer, their carers and healthcare professionals following emergency admission to an acute hospital	Patients were satisfied with their ‘emergency’ care but not the care they received once their initial symptoms had been stabilised. The poorer quality care they experienced was characterised by a lack of attention to their fundamental needs, lack of involvement of their family, poor communication about care plans, and a lack of continuity between primary and secondary care
Chen et al. (2019), UK	Qualitative	Patients (*n* = 15) and carers (*n* = 12)	Age range 40–89	Mixed (unspecified)	Emergency oncology service (via ED or direct admission)	To generate evidence useful for improving services for direct patient benefit, this study explored service users’ views and experiences of emergency admissions and subsequent inpatient care	Participants experienced outstanding inpatient care because of the following: prompt and effective symptom control and stabilisation of acute conditions; continuity of cancer care and coordination between acute and long-term treatment; satisfactory professional-patient communication and information sharing; responsive, motivated, and competent staff; and less restrictive visiting times
Cogo et al. (2020), Brazil	Qualitative	Clinicians (*n* = 12)	N/A	Mixed (unspecified)	ED	To understand nurses’ and physicians’ perceptions of the care of people with cancer who are admitted to the Emergency Department of a general hospital	Three categories emerged: (1) the person with cancer from nurses and physicians’ perspective; (2) comprehensive care of people with cancer or deconfiguration in the emergency department?; and (3) the context of the emergency department and the repercussions on the care of people with cancer. Professionals characterised the need for careful and sensitive care of a person with unpredictable and unfavourable clinical conditions
Hjermstad et al. (2013), Norway	Pre-post/before-and-after	Patients (*n* = 44)	Age range 53–89	Mixed (specified)	ED	To register the reasons for the emergency admissions, to examine symptom intensity upon admission and discharge, and to register the interventions performed during hospitalisation. Patients’ opinions about the emergency admission were also assessed	Respiratory problems, gastrointestinal symptoms, and pain were the most frequent reasons for admissions. Mean pain scores on the Edmonton Symptom Assessment System were reduced by 50% from admission to discharge (*p* < 0.01). Simple interventions such as hydration, bladder catheterisation, and oxygen therapy were most frequent. Nearly one-third of patients would have preferred treatment at another site, provided that the quality of care was similar. Home visits by their family doctor and specialised care teams were perceived by patients as important to prevent hospitalisation
Jelinek et al. (2013)*, Australia	Qualitative	Clinicians (*n* = 83)	N/A	Mixed (unspecified)	ED	To explore the views and experiences of clinicians caring for advanced cancer patients who access the ED	The overarching theme was EDs being ‘caught in the middle’ when providing care to patients with advanced cancer in the physical environment (privacy, noise, lack of information and delay, and lack of defined pathways), the available resources (access block and overcrowding, time pressures, competition with other emergencies, lack of alternatives) and the roles and expectations of the staff providing care (seniority and comfort with dying, views of dying in the ED, expertise, and comfort in caring for those with advanced illness)
Jørgensen et al. (2021), Denmark	Qualitative	Patients (*n* = 8) and carers (*n* = 4)	Age range 52–73	Mixed (unspecified)	Oncological emergency telephone (OET)	To explore patients’ and caregivers’ experience of calling an OET	Patients and caregivers perceived the emergency telephone as a lifeline that they consider calling when the patient’s condition changes from what they understand as normal to what they perceive as abnormal. They would rather call “one time too many than one time too few” if their resources are inadequate to ensure their safety. The tone, attitude, and professional competency of healthcare providers affect patients’ experience of the call
Kaufmann et al. (2020), USA	Qualitative	Patients (*n* = 49)	Mean age 57	Solid tumours	Unplanned acute care (via ED or admission to inpatient solid tumour service)	To explore the viewpoints of patients with cancer who experienced unplanned acute care and describe their recommendations for strategies that may help prevent unnecessary acute care	Themes relating to the decision seek acute care, emotional concerns influencing interactions with patients’ oncology teams, and strategies to avoid acute care were identified. Patients’ recommendations for interventions included anticipatory guidance, peer support, improved triage methods, and enhanced symptom management. Patients preferred options for virtual and home-based outpatient care
Marck et al. (2014)*, Australia	Cross-sectional survey	Clinicians (*n* = 681)	N/A	Mixed (unspecified)	ED	To assess the barriers and enablers as perceived by ED clinicians regarding the care for patients with advanced cancer in the ED. environment. This study aimed to describe findings relevant to caring for dying patients with advanced cancer in the ED	Respondents demonstrated a wide range of views regarding caring for this patient group in ED through free text responses. Although 83.8% found caring for the dying a reasonable demand on their role, the same percentage of clinicians also agreed that the ED is not the right place for end-of-life care in this group. In addition, 64.5% reported that futile treatment is frequently provided in the ED; the main reasons reported were that treatment escalation planning not clearly documented or discussed with the patient and their family. Almost all (94.6%) agreed that advance care plans supported their decisions about caring for dying patients in the ED
Nguyen et al. (2016), Canada	Mixed methods	Patients (*n* = 792)	Age ≥ 70	Mixed (unspecified)	ED	To describe the profile of elderly cancer patients aged 70 years and older who visited the ED of a regional hospital in Québec, Canada, and to explain the medical reasons and factors. determining such visits from the patients’ perspective	The most frequent reasons for attending the ED were respiratory (15.8%), gastrointestinal (13.4%), neurological (8.3%), fever or infection-related (8.3%), and cardiovascular (8.2%) problems. Content analysis of the qualitative data suggested that patients made ED visits mostly when other cancer care services were unavailable or because of a serious life-threatening health condition
Olsson et al. (2021), Sweden	Cross-sectional survey	Clinicians (*n* = 42)	N/A	Mixed (unspecified)	Combined acute oncology-palliative care unit	To investigate the perceptions of HCPs working in a combined acute oncology-palliative care unit regarding the quality of the palliative care received by the patients and how important the care was to the patients	Areas for improvements related to symptom relief, spiritual and existential needs, information, patient participation, continuity of care, care planning, cooperation, and coordination of care were identified; subjective importance scores for these items were higher than corresponding scores for care received
Philip et al. (2018), Australia	Qualitative	Patients (*n* = 19) and carers (*n* = 10)	Mean age 67	Mixed (unspecified)	ED	To explore the experiences and perceptions of EDs held by patients with advanced cancer and their informal caregivers	ED presentations were largely prompted by worsening symptoms or were a means to expedite hospital admission, with many instructed to attend by their health care provider. The experience in the ED was described as a time of anxiety and uncertainty with concerns about communication, the general environment, and delays in the symptom management highlighted. Long waits were common. Despite this, patients described relief at receiving care. Whilst the ED was viewed as a safety net for the health system, many believed advanced cancer patients should have alternative options
Smith et al. (2010), USA	Qualitative	Patients (*n* = 12) and carers (*n* = 7)	Age range 20–89	Mixed (unspecified)	Palliative care (via ED)	To explore the perceptions, experiences, and beliefs of acutely symptomatic patients with terminal illness seen in the ED and their family caregivers	Five distinct themes emerged: (1) unprepared for managing symptoms at home; (2) uncertainty and anxiety; (3) communication is essential; (4) mixed experiences with symptom management; and (5) conflicting perspectives about the purpose of palliative care clinicians in the ED
Waller et al. (2023), Australia	Cross-sectional survey	Patients (*n* = 181)	Age range 19–86	Brain cancer	ED	To examine the perceptions of a sample of outpatients and their accompanying support persons regarding what constitutes optimal care for people with brain cancer presenting to ED	The survey items endorsed as ‘essential’ by participants included that the emergency department team help patients: ‘understand signs and symptoms to watch out for’ (51%); ‘understand the next steps in care and why’ (48%); ‘understand if their medical condition suggests it is likely they will die in hospital’ (47%); ‘ask patients if they have a substitute decision maker and want that person told they are in the emergency department’ (44%); and ‘understand the purpose of tests and procedures’ (41%)
Warrington et al. (2016), UK	Mixed methods	Patients (*n* = 40)	Age range 19–95	Mixed (unspecified)	Acute oncology telephone triage (TT)	To explore patient experiences of admission to the acute oncology service and to analyse the use of the TT system for cancer-related adverse events (and describe: the overall completion and data quality of TT documentation; reported symptoms and their severity; the advice given and the relation between advice and symptom severity)	Patient questionnaires and interviews revealed high satisfaction rates with the streamlined system. Patients reported that the hospital staff knew about their cancer and treatment and felt confident that the staff could deal with their problem. Patients were also well informed about potential side effects of their treatment and how to access the hospital. Although around a third of patients contacted the hospital on the first day, they experienced symptoms, over 50% of patients (most of which were older) had symptoms for up to a week before they sought advice. The most commonly reported symptoms were pain, fever or symptoms of infection, diarrhoea, vomiting, and dyspnoea. The most commonly reported issues which were not listed on the TT form were queries about medications or devices (three calls, 15%) and confusion (three calls, 15%)
Weiland et al. (2015)*, Australia	Cross-sectional survey	Clinicians (*n* = 681)	N/A	Mixed (unspecified)	ED	To add to our limited knowledge of the environmental factors that may affect Australian emergency clinicians’ attitudes to caring for patients with advanced cancer who present to an ED… Additionally, we explored how these clinician attitudes were affected by access to palliative care services, receipt of palliative care education, experience in ED, staff type, ED patient demographic, hospital type, and region. Predictors of frustration in providing optimal care were also explored	They [HCPs] reported overcrowding, noise, lack of time, and privacy as barriers to care. Most (93.3%) agreed/strongly agreed that the dying patient should be allocated private space in ED. 73.6% (451) felt unable to provide a desired level of care to advanced cancer patients in ED. Clinician attitudes were affected by staff type, experience, ED demographic, and hospital type, but not education in palliative care… Integrating palliative care services in ED and redesigning EDs to better match its multifaceted functions should be considered

*ED*, emergency department; *HCP*, healthcare professionals; *N/A*, not applicable; *OET*, oncological emergency telephone; *TT*, telephone triage

^*^Interrelated articles

There was a set of three interrelated articles published as part of the same research project (see Table 2). Qualitative research conducted by Jelinek et al. [22] was preliminary to a large national survey reported by Marck et al. [27] and Weiland et al. [29]. Survey results were reported in two halves; both articles presented primary data analyses, which analysed different sections of the survey for the same participant group.

### Evidence synthesis

Patient experiences of urgent and emergency cancer care were collated into seven major domains of interest: managing emotional distress, palliative care discussions, being treated with compassion and respect, deciding when to seek help, hospital environments, quality of care and communication, and discharge planning. These domains are subsequently described under relevant headings and are broken down into further descriptive sub-domains.

### Managing emotional distress

#### Patient distress and symptom-related anxiety

Three studies that asked patients with cancer and their carers directly about their experiences highlighted managing patient distress as central to the overall experience of emergency cancer care [23–25]. This included one study of advanced cancer patients who had attended an ED, which described the patient experience as ‘a time of anxiety and uncertainty’[25]. All three studies referencing ‘distress’ described patients’ uncertainty surrounding symptoms and anxiety about what symptoms may mean for their prognosis. Symptom-related anxiety was also described within the ‘uncertainty and anxiety’ theme conceptualised by Smith et al. [26] in relation to acutely symptomatic patients admitted via the ED.

#### Carer burden

The burden of supporting someone with cancer experiencing an oncological emergency was documented by three studies [20, 25, 33]. Studies highlighted the burden of responsibility in reporting and managing symptoms by both patients and carers.

#### Palliative care discussions

Eight articles discussed the impact of introducing palliative care to patients in urgent care settings [21, 22, 25–29, 33]. This included two studies which focused the research question on how patients perceived the integration of palliative care within AO services [28, 29]. Smith et al. [26] explored the experiences of patients with incurable cancer who attended an ED, finding conflicting perspectives regarding the impact of introducing ‘palliative care’ to patients. Some patients equated palliative care to end-of-life care and thus felt shocked or unsettled at this introduction in an already distressing scenario. Other patients had been introduced to palliative care before the emergency episode, hence understood that palliative care included a focus on symptom control, which ultimately improved their experience during the emergency episode.

Furthermore, Philip et al. [25] investigated ED experiences of patients with advanced cancer who reported the view that those with advanced cancer should be fast-tracked through, or separated from, the ED. Overall, the literature suggests introducing palliative care during a hospital admission for an oncological emergency can polarise patient experience, with some patients’ experiences improved through the advocacy, psychosocial support, and expertise in pain control that palliative care offers, and others’ negatively impacted by the sudden introduction of the possibility that they might be reaching the ‘end-of-life’. However, regardless of patients having prior knowledge of palliative care or not, ED remains a hectic environment, lacking privacy, and an environment where it can be challenging to discuss and attend to, palliative care needs [22].

### Being treated with compassion and respect

#### Fundamental care

Lack of attention to basic needs in cases of emergency admission was highlighted through the experiences of patients with lung cancer [19]. This topic was also noted by clinicians in another study, specifically about ensuring patient access to food and drink of their preference as a fundamental care need [28].

#### Family and next of kin involvement

Six studies reported the importance to patients of involving their family or next of kin as much or as little as they would like them to be [19, 22, 25, 27, 28, 30]. Relating to the point on palliative care, Marck et al. [27] highlighted a barrier to caring for advanced cancer patients in emergency scenarios when decisions about treatment escalation planning had not been prior discussed with patients’ families. Another study found that next of kin involvement, as a ‘substitute decision maker’, was deemed ‘essential’ to optimal emergency care by patients with brain cancer and their carers [30].

#### Spiritual needs

Olsson et al. [28] noted spiritual needs are important to the patient experience. They found a statistically significant difference between the importance placed by patients on spiritual and existential needs versus the quality of care they felt they received regarding this aspect. There is a paucity of research exploring spiritual needs of patients presenting with oncological emergencies; hence, it is difficult to evaluate the importance of spiritual needs to patients in this context.

#### Privacy and respect

The articles by Marck et al. and Weiland et al. [27, 29], which analysed the same survey data, highlighted the issue of privacy as a barrier to high quality emergency care for patients with advanced cancer. The qualitative accounts from the related qualitative study by Jelinek et al. [22] mirror this view, with limited privacy a barrier to high quality care and optimal patient experiences. Separately, Olsson et al. [28] recognised the importance of treating patients with respect and empathy.

### Deciding when to seek help

Seven articles included in this review discussed findings surrounding how and when patients decide to seek help in an oncological emergency [20, 23–26, 31, 32], whether that be via telephone-delivered emergency cancer care or presenting directly to a service. The decision to seek help appears central to patient experience, as delayed presentation can change the course of emergency care that patients receive. This was clearly evident in one study where more than 50% of patients had symptoms for up to a week before calling the oncology helpline [32].

Qualitative data analysed by Jørgensen et al. [23], highlighted the responsibility that patients and carers feel when considering whether to use telephone-delivered emergency cancer care. The secondary analysis focused on responsibility, finding that patients and carers felt a sense of responsibility in monitoring symptoms, a burden they were relieved of when entrusting the health professional on the phone with making treatment decisions. The study by Kauffmann et al. [24] expanded further on the drivers of accessing unplanned acute cancer care. These included fear of cancer as a driver for treatment-seeking, which was contrasted by hesitancy to access care due to feeling guilty for ‘bothering’ providers. Patients also described ‘waiting out’ symptoms to avoid the ED, with a preference for oncologist involvement in all aspects of care. Patients suggested alternative triage methods such as telehealth, as has been implemented elsewhere [32], to streamline access to urgent and emergency cancer care.

### Hospital environments

Hospital environments were discussed in terms of overcrowding, delayed care, and understaffing. Most pertinent to emergency cancer care, delays, and long waiting times were mentioned in six articles [24–26, 28, 32, 33]. Long waits were common in the ED [25] and, similarly, waiting times did not meet patients’ expectations of an AO service [28]. References to understaffing were made in ED contexts, where findings together indicate a lack of specialist staff for people with cancer, and that advanced cancer patients are generally unable to get the care they need in the ED [21, 27, 29]. Summarising clinician perspectives, Weiland et al. found the core environmental barriers to optimum care provision were overcrowding, noise, lack of time, and privacy [29]. Mirroring this, patients and carers describe the emergency hospital environment as noisy, hectic, and busy [25].

### Quality of care and communication

#### Dealing with the emergency itself

Five studies relayed that acute symptom relief, in general, is a priority in oncological emergencies. Specific symptoms and issues mentioned across all included studies are listed in Table 3.

**Table 3:** Symptoms or issues detailed as requiring acute symptom management during a cancer emergency, extracted from included studies

Symptom or issue	Number of unique studies (*N* = 14)
Pain	7
Breathlessness or respiratory issues	7
Gastrointestinal issues	6
Fever and/or infection	6
Nausea and vomiting	4
Fatigue	3
Skin problems (itching, rash)	2
Neurological (including confusion)	2
Bleeding	2
Lack of sleep	1
Syncope	1
Dizziness	1
Weight loss	1
Loss of appetite	1
Cardiovascular issues	1

How well these acute symptoms are managed is a core component of patient experience in oncological emergencies. Smith et al. [26] describe mixed experiences of acute symptom management for patients requiring palliative care. Whereas, Warrington et al. [32] and Chen et al. [20] found high patient satisfaction with symptom control and stabilisation of acute medical conditions. Further illuminating the patient experience, findings from Bailey et al. [19] found that whilst patients were satisfied with their immediate, ‘emergency’ care, they were not satisfied with the follow-up care they received once their initial symptoms were stabilised.

#### The patient-HCP relationship

Five studies [20, 23–25, 32] reported on patient confidence in staff or the perceived competence of staff during an oncological emergency. In particular, one study of telephone-delivered emergency cancer care indicated that the perceived competence of the clinician impacts their experience, where patients then feel safe and relieved in the emergency scenario [23].

Beyond staff competence, patient-HCP relationships are impacted by how the care team address the patients’ emotional concerns [24], such as their anxiety and uncertainty surrounding what symptoms mean. Tone and attitude are important [23], as is patients’ knowing who is responsible for their care, such that they do not feel ‘lost in the system [25]; a basic level of rapport is valued. Furthermore, Chen et al. [20] noted that patients and carers felt listened to by staff and felt that staff took them seriously, whilst maintaining a positive ‘can-do’ attitude.

#### Communication about care

Communication with patients about their care and what will happen is ‘essential’ to the perceived quality of care. Eight studies discussed this issue [19, 20, 25–28, 30, 32]. The study by Waller et al. [30] focused on elements of optimal care for brain cancer patients presenting to the ED, finding the top two most endorsed items from their suggested list were about communication. These were understanding signs and symptoms to watch out for, and understanding the next steps in care, and why. Chen et al. [20] stated the care patients received was outstanding, wherein they discussed that patients and carers felt well-informed regarding the disease, treatment, and care arrangements, among other factors. Shared-decision making was touched upon in the service evaluation by Olsson et al. [28], highlighting that improvements in information giving and patient participation were needed. They also highlighted care continuity as an area for improvement, which was discussed within five included studies [19, 20, 22, 25, 28]. In particular, Bailey et al. [19] emphasise poor communication with patients about care plans and a discontinuity between primary and secondary services as factors that negatively impact patient experiences of emergency cancer care.

### Discharge planning

Planning for patient discharge post-emergency also affected patient experiences. This issue included patient and carer preparedness for managing symptoms and treatment at home, as well as knowing what symptoms to look out for. As aforementioned, knowing what signs and symptoms to watch out for was the most highly endorsed item contributing to optimal care of patients with brain cancer [30], according to patients and carers. Kauffmann et al. [24] further found that patients who have experienced emergency cancer care advocate for more supportive interventions to enable patients to self-manage at home. This could be done through provision of anticipatory guidance, including symptom management education and action planning for acute events.

## Discussion

This review has drawn together for the first time the core issues of importance to the experiences of people with cancer, and their carers, when seeking help for oncological emergencies. These issues were managing emotional distress, palliative care discussion, being treated with compassion and respect, deciding when to seek help, hospital environments, quality of care and communication, and discharge planning. These areas of importance are summarised based on findings from 15 research papers and from 14 unique studies. Several of these issues are enmeshed with the cancer experience as a whole, such as the nature of hospital environments and the quality of communication. There were, however, issues captured unique to emergency cancer care, such as patient distress resulting from uncertainty, deciding when to seek help, and the impact of mentioning ‘palliative care’ in urgent and emergent scenarios.

Fifty percent of the articles captured the importance of sensitively managing patients’ palliative care needs during an oncological emergency [21, 22, 25–29, 33], under the major domain of ‘being treated with compassion and respect’. Overarchingly, patients’ experience during emergencies, particularly when presenting to the ED, were impacted by whether or not they had been introduced to palliative care before, and their understanding of its benefits. For example, patients in one study welcomed pain management offered by palliative care specialists, over and above pain management offered by ED clinicians [26], subsequently improving acute symptom management and overall experience. In contrast, some patients were shocked by the introduction of palliative care during an already distressing scenario, due to its conflation with end-of-life care. International guidance recommends early palliative care involvement for patients with uncontrolled symptoms and the integration of specialist supportive and palliative care into treatment plans from cancer diagnosis onwards [35]. In addition, ensuring people with advanced cancer are given the opportunity to discuss advanced care planning and contribute to treatment escalation decisions during periods of stability might avoid unfamiliar emergency care clinicians having to broach difficult conversations in a hectic, often inappropriate, environment [22, 27].

Within the domain ‘deciding when to seek help’, options for remote care to improve access to AO services were emphasised, with two studies reporting on patient experiences of telephone-delivered emergency cancer care [23, 32] and one study suggesting telehealth as a solution to reduce ED presentations [24]. Beyond the application of telehealth for assessing acute cancer-related symptoms, other research has explored the issue of access and care coordination in urgent and emergent cancer care, noting the potential for web-based symptom self-reporting, urgent cancer care clinics, and alternative models of care, such as telephone triage [34]. Whilst these appear to be effective in reducing ED visits and hospitalisations, there remains no clear model of best practice [7], and little is known about how, in particular, web-based triage is perceived by patients during an emergency oncologic episode. Access to care is critically important in cancer-related emergencies; beyond attempts to streamline models of access, there is a paucity of research investigating other barriers to accessing emergency cancer care. These may include access for those with learning difficulties, different language needs, audio-visual impairments, as well as other socio-demographic factors which can contribute to delayed presentation, and potentially impact patient experiences of the emergent care that follows.

This review was conducted as part of developing a PREM [11, 15] for use in the context of AO services. This measure is intended to be utilised to capture patient experiences of emergency cancer care, such that the outcomes that matter to patients and their families can be analysed and improved [10]. There are several existing PREMS for use within ED [35–38]; however, none fully address the specific issues related to patients with cancer experiencing an oncological emergency identified in this review, such as their preparedness for help-seeking via AO telephone triage, their interaction with palliative care professionals, discharge planning, and the information given to them of symptoms to watch for that should trigger future contact with the AO team. Similarly, there are PREMs available for cancer patients, such as the National Cancer Patient Experience Survey [39] and the Consumer Assessment of Healthcare Providers and Systems Programme (CAHPS) of the Agency for Healthcare Research and Quality (AHRQ) [40], but these do not include items specific to AO. The next steps in the development of the AO PREM are to use the domains and subdomains collated herein, alongside interviews with patients, to further develop the measure. This will be followed by a feasibility study within an AO service in the UK.

### Limitations

There are various terms used to describe emergency cancer care and different service delivery models, in the UK and internationally. Efforts were made to use broad terminology to conduct the initial database searches for this review, including search terms for ambulatory care. We recognise that some articles, particularly from researchers outside the UK, may not have been retrieved due to differences in terminology, although articles from the USA, Canada, Australia, Denmark, Sweden, and Brazil were retrieved. Only studies published in English were included due to resource constraints. Grey literature and unpublished works were not included in the review.

## Conclusions

This review synthesised existing literature on patient experiences of emergency cancer care, highlighting core domains of the patient experience impactful for patients and their families. Deciding when to seek help and discussing palliative care needs with patients in the context of an oncological emergency were covered in detail. These two issues were more specific to emergency care, compared to other domains of experience which related to cancer care more generally. The results from this study will be used to develop a PREM, such that specialist urgent and emergency cancer care services are enabled to evaluate and continuously improve.

## Supplementary Information

Below is the link to the electronic supplementary material.

Supplementary file1 (DOCX 27 KB)

## Data Availability

The datasets used and analysed during the current study are available from the corresponding author on reasonable request.

## Abbreviations

ED: Emergency Department
HCP: Health care professional
NHS: National Health Service
UK: United Kingdom (UK)
USA: United States of America
PREM: Patient-reported experience measure
AO: Acute oncology

## Declarations

### Disclaimer

The views expressed in this publication are those of the authors and not necessarily those of the NHS, the NIHR or the Department of Health and Social Care.
