# Supplementary material for: Mixed methods scoping review of patients’ experiences of urgent and emergency cancer care
Source: Support Care Cancer. 2025 Feb 21;33(3):208. doi: 10.1007/s00520-025-09245-8 (PMC11842481; doi:10.1007/s00520-025-09245-8)
Supplement: Supplementary file 1 — Supplementary file1 (DOCX 27 KB) [file 520_2025_9245_MOESM1_ESM.docx]

## Additional file 1 – Full search terms (all databases)

### MEDLINE, CINAHL, PsycINFO

| Component |  |  |
| --- | --- | --- |
| Population | Patients with cancer | AB ( (cancer* N3 patient*) OR cancer* OR malignan* OR oncolog* OR haematolog* OR hematolog* OR neoplas* OR tumor* OR tumour* OR metasta* OR chemo* OR ( (anti-cancer OR anticancer OR active) N1 treatment) OR radiotherap* OR immunotherap* ) OR TI ( (cancer* N3 patient*) OR cancer* OR malignan* OR oncolog* OR haematolog* OR hematolog* OR neoplas* OR tumor* OR tumour* OR metasta* OR chemo* OR ( (anti-cancer OR anticancer OR active) N1 treatment) OR radiotherap* OR immunotherap* ) |
| Context | Acute oncology | AB (“urgent care” OR “acute oncology” OR “acute care” OR (emergency N3 (care OR department* OR service*) ) OR ambulatory OR helpline OR (telephone N3 triage*) ) OR TI (“urgent care” OR “acute oncology” OR “acute care” (emergency N3 (care OR department* OR service*) ) OR ambulatory OR helpline OR (telephone N3 triage*) ) OR  (MH "Ambulatory Care") OR (MH "Emergencies") OR (MH "Emergency Service, Hospital") OR (MH "Emergency Treatment") OR (MH "Emergency Medicine") OR (MH "Emergency Medical Services") OR (MH "Critical Care") OR (MH "Emergency Patients") OR (MH "Emergency Care") OR (DE "Emergency Medicine") OR (DE "Emergency Services") |
| Concept | Experience of care | AB ( (patient* N3 (experience* OR satisfaction OR perception* OR view* OR opinion*) ) OR (caregiver* N3 (satisfaction OR perception* OR view* OR opinion*) ) OR (treatment* N3 (experience* OR satisfaction OR perception* OR view* OR opinion*) ) OR care N3 quality ) OR TI ( (patient* N3 (experience* OR satisfaction OR perception* OR view* OR opinion*) ) OR (caregiver* N3 (satisfaction OR perception* OR view* OR opinion*) OR (treatment* N3 (experience* OR satisfaction OR perception* OR view* OR opinion*) ) OR care N3 quality ) |

Search fields: title (TI), abstract (AB), subject headings (MH), subjects [exact] (DE).

Limiters: English language, peer-reviewed, human.

### Web of Science

| Component |  |  |
| --- | --- | --- |
| Population | Patients with cancer | (cancer* NEAR/3 patient*) OR cancer* OR malignan* OR oncolog* OR haematolog* OR hematolog* OR neoplas* OR tumor* OR tumour* OR metasta* OR chemo* OR ( (anti-cancer OR anticancer OR active) NEAR/1 treatment) OR radiotherap* OR immunotherap* (Topic) |
| Context | Acute oncology | “urgent care” OR “acute oncology” OR “acute care” OR (emergency NEAR/3 (care OR department* OR service*) ) OR ambulatory OR helpline OR (telephone NEAR/3 triage*) (Topic) |
| Concept | Experience of care | (patient* NEAR/3 (experience* OR satisfaction OR perception* OR view* OR opinion*) ) OR (caregiver* NEAR/3 (satisfaction OR perception* OR view* OR opinion*) ) OR (treatment* NEAR/3 (experience* OR satisfaction OR perception* OR view* OR opinion*) ) OR care NEAR/3 quality (Topic) |

Search fields: topic (title, abstract, keywords and keywords+).

Limiters: English language.

### EMBASE (Ovid)

| Component |  |  |
| --- | --- | --- |
| Population | Patients with cancer | ((cancer* adj3 patient*) OR cancer* OR malignan* OR oncolog* OR haematolog* OR hematolog* OR neoplas* OR tumor* OR tumour* OR metasta* OR chemo* OR ((anti-cancer OR anticancer OR active) adj1 treatment) OR radiotherap* OR immunotherap*).ti,ab. |
| Context | Acute oncology | (urgent care OR acute oncology OR acute care OR (emergency adj3 (care OR department* OR service*)) OR ambulatory OR helpline OR (telephone adj3 triage*)).ti,ab. |
| Concept | Experience of care | ((patient* adj3 (experience* OR satisfaction OR perception* OR view* OR opinion*)) OR (caregiver* adj3 (satisfaction OR perception* OR view* OR opinion*)) OR (treatment* adj3 (experience* OR satisfaction OR perception* OR view* OR opinion*)) OR (care adj3 quality)).ti,ab. |

Search fields: title (ti) and abstract (ab).

Limiters: English language.
